# Deciphering the Heterogeneity of Cancer-Associated Fibroblasts in Prostate Cancer: From Stromal Biology to Clinical Translation

**DOI:** 10.3390/cancers18101600

**Published:** 2026-05-14

**Authors:** Ho Trong Tan Truong, Whi-An Kwon, Hyeong Jung Woo, Minseok S. Kim, Nhu Quang Tran, Jae Young Joung

**Affiliations:** 1 Department of Urology, Cho Ray Hospital, Ho Chi Minh City 700000, Vietnam; truonghotrtan@gmail.com (H.T.T.T.); trannhuquang1302@gmail.com (N.Q.T.); 2 Department of Urology, Hanyang University College of Medicine, Myongji Hospital, Goyang 10475, Republic of Korea; 3 Research Institute of Precision Medicine and Geroscience, Myongji Medical Foundation, Goyang 10475, Republic of Korea; 4 Department of New Biology, DGIST, Techno Jungang-Daero 333, Daegu 42988, Republic of Korea; jjwoo96@dgist.ac.kr (H.J.W.); kms@ctcells.com (M.S.K.); 5 CTCELLS Inc., Seoul 06307, Republic of Korea; 6 Department of Urology, Urological Cancer Center, National Cancer Center, Goyang 10408, Republic of Korea

**Keywords:** prostate cancer, cancer-associated fibroblasts, tumor microenvironment, castration-resistant prostate cancer, spatial transcriptomics, single-cell analysis, liquid biopsy, fibroblast activation protein

## Abstract

Prostate cancer (PCa) progression and treatment resistance are driven by tumor-intrinsic mechanisms and adaptive remodeling of the tumor microenvironment, in which cancer-associated fibroblasts (CAFs) play a crucial role. Although CAF biology is increasingly recognized, a major translational gap remains: CAFs are highly heterogeneous, and comprise distinct functional states with divergent effects on disease progression, immune regulation, and therapeutic resistance. To bridge this gap, we synthesize evidence from single-cell and spatial transcriptomic studies, tissue-based pathology, liquid biopsy assays, and molecular imaging to construct an evidence-tiered, decision-oriented translational framework that connects stromal mechanisms, translational measurement strategies, and therapeutic interventions in PCa. Single-cell and spatial transcriptomic analyses have consistently identified multiple CAF programs, including matrix-remodeling, inflammatory, immunoregulatory, antigen-presenting, and therapy-imprinted states, each with distinct functional outputs and clinical correlates. Tissue-based readouts, including reactive stromal grade (RSG) and fibroblast activation protein (FAP) immunohistochemistry, provide practical proxies for stromal activation and correlate with disease-specific mortality and imaging phenotypes. Circulating CAFs (cCAFs) represent an emerging liquid biopsy modality for longitudinal stromal monitoring, although technical standardization is required before clinical implementation. FAP-targeted PET imaging and emerging dual prostate-specific membrane antigen (PSMA)/FAP-targeted theranostic strategies provide noninvasive tools for patient selection and response assessment, particularly in PSMA-discordant or tracer-heterogeneous disease. Androgen receptor (AR)-targeted therapy can reprogram stromal states toward resistance-promoting circuits, highlighting the dynamic and plastic nature of the CAF compartment. A state-based CAF framework organizes stromal biology into testable translational hypotheses rather than immediate clinical standards. RSG and FAP-based tissue or imaging readouts are practical markers of stromal activation, whereas spatial CAF-immune signatures and cCAF assays remain investigational and require assay harmonization and prospective validation. Future trials should pre-specify stromal biomarkers as enrichment or pharmacodynamic variables when matched to the intervention and should avoid treating CAFs as a uniform therapeutic target.

## Simple Summary

Prostate cancer (PCa) progression is shaped not only by malignant epithelial cells but also by cancer-associated fibroblasts (CAFs) in the surrounding stroma. CAFs remodel tissue architecture, provide survival signals, suppress antitumor immunity, and promote treatment resistance. Importantly, CAFs are not a uniform population; they occupy multiple functional states with distinct biological roles that evolve across disease stages and under therapeutic pressure. This review synthesizes evidence that CAF state diversity contributes to local invasion, immune exclusion, metabolic adaptation, and castration resistance in PCa. We further examine practical clinical tools for assessing CAF activity in clinical settings, including tissue-based stromal grading, transcriptomic and spatial signatures, circulating CAFs as liquid biopsy surrogates, and fibroblast activation protein-targeted imaging with theranostic potential. Finally, we propose a stromal precision medicine framework that links dominant CAF programs to mechanism-aligned interventions and biomarker-guided trial designs, providing a roadmap for translating stromal biology into prospectively testable therapeutic hypotheses.

## 1. Introduction

Prostate cancer (PCa) remains clinically challenging because its pathogenesis and resistance mechanisms reflect tumor-intrinsic alterations and dynamic interactions within the tumor microenvironment (TME). The TME is a key determinant of cancer progression and therapeutic vulnerability [1]. Within this ecosystem, stromal components, particularly cancer-associated fibroblasts (CAFs), are active participants rather than passive bystanders. CAFs actively remodel the extracellular matrix (ECM), thereby altering tissue stiffness and transport. Additionally, they secrete cytokines, chemokines, growth factors, and metabolites that reprogram tumor cell plasticity and enhance stress tolerance [2].

The importance of stromal biology in the prostate is therefore well established. Early studies demonstrated that epithelial activity is tightly regulated by the surrounding stroma and that activated prostate stromal cells can directly promote carcinogenesis and disease progression rather than responding passively to tumorigenesis [3,4]. Clinically, this concept is reflected in reactive stroma, a histological process characterized by fibroblast activation and ECM remodeling that appears early and evolves alongside tumor development [5]. Reactive stromal grade (RSG), assessed in diagnostic biopsies, has been associated with PCa mortality in population-based studies, and its prognostic value is strengthened when combined with adverse vascular features, including lymphovascular invasion (LVI) [6,7]. These data indicate that stromal state is biologically meaningful and clinically observable.

However, a key gap remains: CAFs are not uniform. Single-cell and spatial profiling consistently identify fibroblasts in multiple transcriptional and functional states, including inflammatory, myofibroblastic, and immunoregulatory programs, whose distribution varies across disease stages and therapeutic exposures [8]. Importantly, CAF biology evolves dynamically during progression. Therapies can alter the stromal environment and promote adaptive resistance mechanisms. Androgen receptor (AR)-targeted therapy, including conventional androgen deprivation therapy (ADT) and next-generation antiandrogen treatments, can reprogram the stroma and promote myofibroblast-like CAFs that support castration resistance through specific ligand-receptor interaction circuits [9]. Beyond transcriptional flexibility, metabolically specialized CAF states may directly promote immunosuppression. For instance, iron-enriched CAF programs have been observed to induce an immunosuppressive phenotype in PCa tissue [10]. These findings reveal that the reversibility or persistence of therapy-induced CAF states remains poorly defined, as does how they can be therapeutically targeted without promoting a more aggressive TME.

Advances in CAF biology have improved patient stratification. Circulating CAFs have been identified and analyzed in metastatic hormone-sensitive prostate cancer (mHSPC), suggesting that stromal states can be monitored through liquid biopsy-based approaches in addition to tumor genomics [11]. Tissue assays using fibroblast activation protein (FAP) and FAP inhibitor (FAPI) positron emission tomography (PET) imaging are promising noninvasive approaches for mapping fibroblast activation in vivo. This may be particularly relevant in castration-resistant prostate cancer (CRPC), including metastatic CRPC (mCRPC), where theranostic applications could provide clinical advantage. However, uncertainties remain regarding its specificity, context dependence, and overall clinical utility across disease phenotypes [12,13].

The novelty of this review is not to propose another CAF nomenclature, but to organize PCa CAF biology into an evidence-tiered translational framework. We specifically distinguish: (i) marker-defined CAF detection from state-level functional inference, (ii) physical ECM-mediated exclusion from chemokine/myeloid-driven immune exclusion, (iii) selective depletion from stromal normalization or reprogramming, and (iv) clinically established readouts from exploratory tools, such as cCAFs and spatial signatures.

## 2. CAFs in Prostate Cancer: Definition, Origins, and State Diversity

### 2.1. Stromal Regulation and Reactive Stroma in the Prostate

The prostate is highly dependent on stromal support to maintain tissue homeostasis. Stromal compartments actively regulate normal prostate physiology and are reprogrammed during tumorigenesis [14]. Traditional stromal -epithelial studies have demonstrated that stromal signaling can guide epithelial proliferation, differentiation, and malignancy, providing a biological basis for the role of stromal alterations in PCa progression [3,4]. In clinical specimens, this concept is exemplified by reactive stroma, which involves fibroblast activation, ECM remodeling, and alterations in paracrine signaling networks [5,15].

RSG provides a practical method for quantifying this process in tissue sections. Reactive stromal features are not merely descriptive; they are robustly associated with clinically meaningful outcomes. Quantitative assessment of stromogenic regions and the RSG in PCa tissues has been associated with disease aggressiveness and PCa-specific mortality [7,16]. This prognostic value may increase when reactive stroma is assessed together with adverse vascular features, including lymphovascular invasion, suggesting that stromal activation and vascular invasion collectively define a high-risk TME [6,7]. These observations underpin the fundamental premise of this review: fibroblast activation states are integral to disease biology and can be measured in routine pathological assessment [6,7,16,17].

### 2.2. Practical Criteria to Define CAFs and Limitations of Marker Panels

CAFs are typically characterized as activated fibroblast-lineage stromal cells located within or in proximity to tumor tissue. They influence tumor progression by remodeling the ECM and secreting signaling molecules [2,18]. In practice, CAF identification depends on combinatorial marker panels rather than a single definitive marker. Commonly utilized positive markers include FAP, αSMA, PDGFRβ, collagen I, and fibronectin, together with exclusion markers for epithelial, endothelial, and immune lineages [2,19]. This operational definition is extensively used owing to its feasibility in immunohistochemistry and compatibility with transcriptomic profiling [2,19].

However, marker-based definitions have certain limitations. First, frequently used markers are not CAF-specific across various contexts. For instance, αSMA-dominant markers preferentially capture contractile myofibroblast-like programs and may underrepresent inflammatory or immunoregulatory fibroblast states [2,19]. FAP is strongly associated with activated fibroblasts and has translational relevance; however, its expression varies by disease stage and microenvironmental context and does not independently resolve functional diversity [2,19]. Second, marker expression is dynamic. Profiling studies consistently indicate that fibroblast programs vary with stage, local environment, and treatment exposure, indicating that identical marker panels may capture different CAF compositions across cohorts [9,20,21,22]. Third, no broadly accepted standard CAF marker panel has been established. Studies often apply different combinations of markers and exclusion criteria according to tissue availability, analytical platform, and research objectives [23]. Consequently, the operational definition of CAFs and the classification of their subtypes can vary substantially between studies. This lack of standardization complicates direct comparison and reproducibility across datasets and limits efforts to define the clinical significance of specific CAF subpopulations.

For a review centered on clinical relevance, CAFs should be characterized using integrated multimodal approaches that combine marker panels with transcriptomic and spatial analyses and are interpreted within the appropriate biological context. This strategy reduces overgeneralization from narrowly defined subsets and aligns with emerging multi-omic and spatial profiling methods [2,19]. Table 1 summarizes commonly used marker panels, the CAF programs they preferentially capture, and their principal blind spots.

### 2.3. CAF States and Functional Programs

Evidence from multiple tumor types indicates that CAFs are heterogeneous and are more accurately characterized as a spectrum of states rather than as a single cell type [2,24]. Mechanistic studies show that microenvironmental cues can direct fibroblasts toward inflammatory or myofibroblastic phenotypes through distinct signaling pathways, illustrating how state diversity is dynamically generated and maintained [25]. This provides a valuable conceptual framework for PCa, wherein multiple CAF programs are increasingly recognized rather than presumed [2,19].

Operationally, we define a CAF state as the concordance of a multi-gene transcriptional program, a dominant functional output, and spatial or orthogonal validation linking that program to a defined tissue niche.

In PCa, single-cell and integrative transcriptomic analyses have identified multiple fibroblast states and proposed state-associated gene signatures linked to clinical outcomes [20,21]. Although nomenclature varies across datasets, the primary functional axes are generally consistent: matrix-remodeling and contractile programs, inflammatory and secretory programs, and immunoregulatory programs that influence immune-cell recruitment and function [19,20,21].

Within the immunoregulatory axis, antigen-presenting CAF (apCAF)-like states are characterized by MHC class II/CD74 expression, usually without the complete costimulatory machinery required for professional antigen presentation [26,27]. Therefore, MHC-II positivity in stromal cells should not be interpreted as immunostimulatory without co-registered CD4/CD8/Treg phenotyping and costimulatory-marker assessment. Although the prevalence of apCAFs in PCa remains uncertain, their identification challenges the assumption that all CAF states are immunosuppressive, a distinction with important implications for spatial immune profiling and immunotherapy trial design [28,29]. The functional interpretation of apCAF-like states remains controversial and context-dependent [30]. In the absence of professional costimulatory signals, such as CD80/CD86, MHC-II-mediated antigen presentation by stromal cells may render CD4^+^ T cells anergic rather than activated [26]. Recent evidence further suggests that CD4^+^ T-cell engagement by apCAF-like cells can preferentially expand regulatory T cells, repositioning these stromal niches as potential mediators of immune tolerance rather than effector immunity [31].

This functional duality implies that apCAF-like quantification alone is insufficient; the phenotype and functional state of neighboring T cells must be assessed in the same spatial context to determine functional polarity [32]. Resolving these ambiguities is precisely why state-level characterization is required beyond simple cell enumeration [33]. Importantly, these states are not merely descriptive. They link fibroblast biology to quantifiable phenotypes, including ECM architecture, tissue stiffness, cytokine gradients, and immune exclusion, as discussed in subsequent sections [2,19]. In PCa, apCAF should be framed as a cross-tumor-derived hypothesis rather than a validated prostate-specific CAF subtype. Available data support spatial evaluation of MHC-II/CD74-positive stromal niches with co-registered CD4/CD8/Treg phenotypes, but not standalone clinical interpretation.

Therapy adds further complexity. AR-targeted therapy can remodel stromal conditions and enhance CAF programs that facilitate castration resistance, thereby highlighting that CAF composition is dynamic and therapeutically modifiable rather than static [9]. Additionally, metabolically specialized CAF programs correlate with immunosuppressive phenotypes, implying that metabolic reprogramming may define clinically relevant stromal states [10,34]. Together, these studies indicate that state-based classification of CAF biology is essential for developing mechanistically rational therapeutic strategies in PCa [2,9,10].

### 2.4. Spatial Organization and Stage-Dependent Changes

CAF function depends not only on state identity but also on spatial organization within the TME. Spatial profiling indicates that fibroblast programs correspond to local neighborhoods near glands, vasculature, immune infiltrates, and other microenvironmental features, potentially affecting gradients in growth signals, transport, and immune access [22]. This spatial organization explains why similar marker patterns may have different biological and clinical implications depending on cellular location and intercellular interactions [22].

The composition of CAFs varies across disease stages. In early disease, reactive stroma reflects tissue-level activation. In advanced disease and after treatment exposure, CAF states shift toward programs associated with therapy resistance and immune suppression [7,9,10].

**Figure 1:**
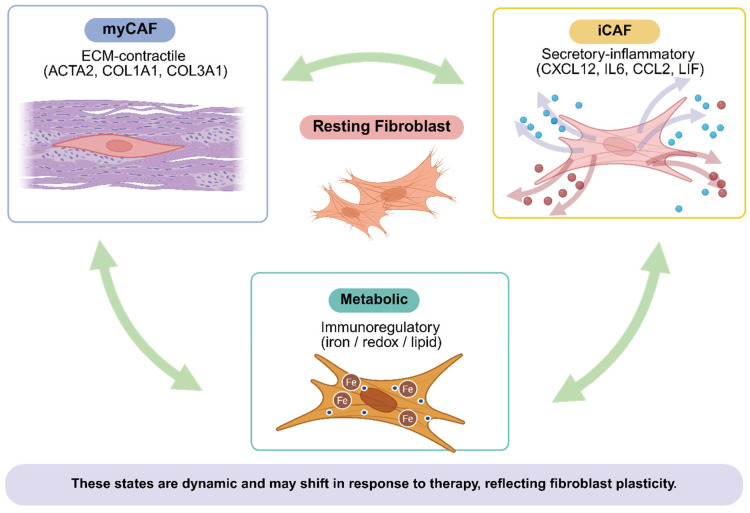
Conceptual framework of dynamic cancer-associated fibroblast (CAF) states in prostate cancer. The labels align commonly used scRNA-seq nomenclature with clinically measurable readouts; state assignment requires co-expression patterns and spatial context, rather than single-marker interpretation. apCAF-like (HLA-DRA, CD74, MHCII^+^) and FAP-enriched activated stroma represent additional clinically distinct programs not depicted in the three-state schematic. Created in BioRender. Kwon, W. (2026) https://BioRender.com/ev16p10 accessed on 2 May 2026.

For the remainder of this review, we use a state-based framework organized around three core functional axes: matrix-remodeling and contractile programs, inflammatory and secretory programs, and therapy- or context-imprinted programs that emerge during progression and treatment [9,19,20,21,22]. Within and alongside these axes, we distinguish apCAF-like programs, immunoregulatory and metabolic programs, and FAP-enriched activated stroma as clinically distinct states warranting separate translational consideration because of their specific measurement strategies and therapeutic implications (Figure 1; Table 2). This evidence-tiered organization supports future biomarker development and trial design, with spatial and stage-specific CAF composition prospectively characterized rather than assumed to be uniform across disease contexts.

## 3. CAF Programs in Tumor Progression and Local Invasion

CAFs facilitate PCa progression through a limited set of core programs consistently observed across models and clinical datasets. These programs influence the physical properties of tissues, survival-signal availability, and the capacity of tumor cells to alter their state under stress. Furthermore, they modulate the interactions between cancer cells and the immune and vascular compartments. Reactive stroma and tumor stroma in PCa therefore provide a valuable framework in which CAF activity is most accurately characterized as the coordinated remodeling of structural and signaling components, not as the effect of a single pathway [2,5,15,18].

### 3.1. ECM Remodeling and Tissue Mechanics

Continuous ECM remodeling is a hallmark of CAF activation. Reactive stroma is characterized by changes in the collagen-rich matrix, stromal composition, and wound-like architecture that may develop alongside tumor progression [5,15]. Beyond collagen, specific stromal ECM proteins, including asporin, have been implicated as microenvironmental regulators in PCa [64]. These matrix modifications are important as they do not merely accompany cancer; they can alter tumor-cell behavior and tissue-level function. Foundational studies of stromal-epithelial interactions showed that stromal programs can influence epithelial phenotype [3,4], and subsequent PCa studies linked stromal remodeling to clinically meaningful endpoints, including PCa-specific mortality [7,16].

Mechanistically, matrix remodeling influences cancer progression by altering tissue mechanics, establishing collective-invasion pathways, and restricting diffusion of oxygen, nutrients, and therapeutics, with downstream consequences for epithelial plasticity and stress-tolerant phenotypes [2,5,15,18,65].

This therapeutic framework requires important qualifications [36]. Dense desmoplasia is not uniformly permissive for progression. In selected contexts, a fibrotic capsule may physically constrain vascular invasion and systemic dissemination [66]. Preclinical evidence and clinical correlates suggest that aggressive ECM dissolution can paradoxically release tumor cells into the vasculature, a mechanistic concern reinforced by the failure of hyaluronidase-based strategies in phase III settings [67]. Therefore, ECM-targeting strategies should be evaluated with metastasis-related endpoints alongside delivery surrogates, particularly in disease phenotypes in which stromal density correlates with organotropic constraints rather than immune exclusion alone [36]. Recognizing this duality does not negate the rationale for stromal normalization; rather, it refines it [68]. These effects help explain how reactive stroma correlates with disease aggressiveness and why stromal strategies often focus on normalizing, reprogramming, or circumventing matrix-driven barriers rather than solely removing fibroblasts [15,18]. Accordingly, RSG and ECM-rich phenotypes warrant prospective evaluation as candidate enrichment or stratification variables in trials targeting matrix remodeling, rather than collection only as exploratory correlates after enrollment.

### 3.2. Paracrine Growth and Survival Signaling

Beyond matrix remodeling, CAFs provide tumor cells with diverse paracrine signals. Studies of prostate tumor-stroma interactions have described reciprocal signaling loops that promote tumor growth and influence tumor-cell differentiation states [69]. Reviews of reactive stroma similarly emphasize that CAFs provide growth factors and cytokines that support survival, motility, and adaptation, whereas tumor cells reciprocally sustain fibroblast activation [5,15]. This reciprocal structure holds significance, as it elucidates the rationale behind the continued existence of CAF programs, even in instances where the initiating insult undergoes changes.

A clear example of stromal-immune-tumor signaling was demonstrated in prostate CAF models, showing that CAFs recruit monocytes and promote an M2 macrophage phenotype, whereas M2 macrophages subsequently enhance CAF reactivity. This synergistic cycle amplifies tumor-cell motility and promotes tumor progression [39]. The study emphasizes stromal-derived factor signaling as a key driver of cellular recruitment and polarization, underscoring the ability of CAFs to remodel the TME by regulating cellular entry and behavior within tumors [39]. In parallel, the CXCL12/CXCR4 axis has been implicated in coordinating tumor-microenvironment interactions relevant to progression, supporting the view that chemokine signaling constitutes a functional component of CAF biology rather than a secondary consequence [70].

Paracrine signaling further links CAFs to therapeutic responses. One principal mechanism involves extracellular vesicle-mediated cargo transfer. In PCa, CAF-derived exosomes containing miR-423-5p reduce chemosensitivity and enhance taxane resistance by targeting GREM2 through a TGF-β-linked pathway; inhibition of TGF-β signaling partially mitigates this effect [71]. This finding provides a concrete link between a CAF program, a clinically relevant phenotype, and verifiable intervention point [71]. These findings support prospective evaluation of resistance profiling alongside tumor genomics in patients progressing on systemic therapy, and CAF-derived exosomal signatures warrant investigation as pharmacodynamic resistance biomarkers [18,40].

### 3.3. Plasticity, Epithelial–Mesenchymal Transition (EMT)-Related Programs, and Stress Tolerance

Local invasion in PCa is strongly influenced by the capacity of tumor cells to change phenotype under microenvironmental pressure. CAFs contribute by delivering signals that promote epithelial-mesenchymal transition-related pathways, stemness-associated traits, and resistance to stressors, such as detachment and therapeutic interventions [2,5]. In PCa, stromal induction of miRNA remodeling is associated with these phenotypes [72]. A focused review of miR-1247 describes stromal-driven downregulation of this miRNA as a pathway that increase malignancy, emphasizing its connections to EMT, invasion, anoikis resistance, and reduced chemosensitivity [73]. These data emphasize that CAF-driven plasticity extends beyond a mere transcriptional concept and possesses functional implications that can be quantified in invasion assays and treatment-response models [73].

CAF-mediated stress tolerance is further reinforced by metabolic adaptation. A substantial literature on CAF metabolism elucidates how CAFs support tumor cells through metabolic reprogramming, thereby promoting aggressive phenotypes and survival under stress [34]. Hypoxia served as an additional enhancer. Hypoxia-driven signaling and exosome-mediated communication have been studied as mechanisms that facilitate PCa progression and reinforce microenvironmental adaptation, including changes that may promote invasion and resistance to therapy [65]. These factors frequently operate in conjunction. Hypoxia elevates selective pressure, CAF programming offers survival cues, and plasticity pathways enable tumor cells to utilize these signals.

Finally, therapy may further amplify these dynamics by altering CAF states. AR-targeted therapy can induce stromal reprogramming that promotes castration resistance through specific intercellular communication pathways, indicating that treatment exposure can actively shape CAF-driven plasticity [9].

Mechanistically, exposure to ADT and AR-pathway inhibitors can reshape stromal states through fibroblast AR suppression and activation of IL-6/STAT3- and NF-κB-linked inflammatory programs. CAF-derived NRG1/HER3 and FGF/FGFR ligand-receptor circuits have also been implicated in androgen-independent epithelial proliferation [9,52]. These pathways provide candidate entry points for combination strategies with AR-pathway inhibition.

This concept is fundamental to understanding the emergence of aggressive phenotypes despite effective initial tumor control. Stromal biomarkers of plasticity, including EMT-linked CAF signatures and therapy-induced state shifts, warrant prospective evaluation as candidate risk-stratification variables and as rational triggers for testing combination interventions before resistance programs become entrenched [9,18].

### 3.4. Angiogenesis, Transport, and Drug Penetration

CAF programs influence local progression by changing cancer-cell state and shaping vascular biology and transport. Reviews of reactive stroma describe how activated fibroblasts support angiogenesis and alter the microvascular environment, contributing to a tissue ecosystem that favors tumor growth and dissemination [5,15]. Simultaneously, stromal remodeling may generate physical barriers. Dense ECM and altered interstitial properties can impede drug penetration and contribute to regional hypoxia, both of which can select for treatment-resistant tumor-cell phenotypes and reduce therapeutic efficacy [5,18,65].

A transport-focused perspective helps integrate observations that may otherwise appear disconnected. First, barriers and gradients provide a mechanistic link between stromal activation observed in pathology and clinical outcomes, because they can influence tumor evolution over extended periods [5,7,16]. Second, this perspective explains why stromal targeting is often regarded as a means to enhance delivery and response, rather than solely to eradicate a cell population [15,18]. Mapping fibroblast activation and stromal architecture in vivo using FAP PET, spatial transcriptomics, or quantitative RSG could be pre-specified as a baseline exploratory or pharmacodynamic assessment in trials where stromal density may limit drug delivery or immune access [18].

In summary, CAF-driven ECM remodeling, paracrine signaling, plasticity support, and transport effects form a coherent set of progression mechanisms. These mechanisms provide the foundation for the subsequent section, which examines how CAF states interact with immune and metabolic circuits and how these interactions contribute to metastatic niche formation [10,34,39,65].

## 4. CAF Crosstalk with Immunity, Metabolism, and Metastatic Niches

**Figure 2:**
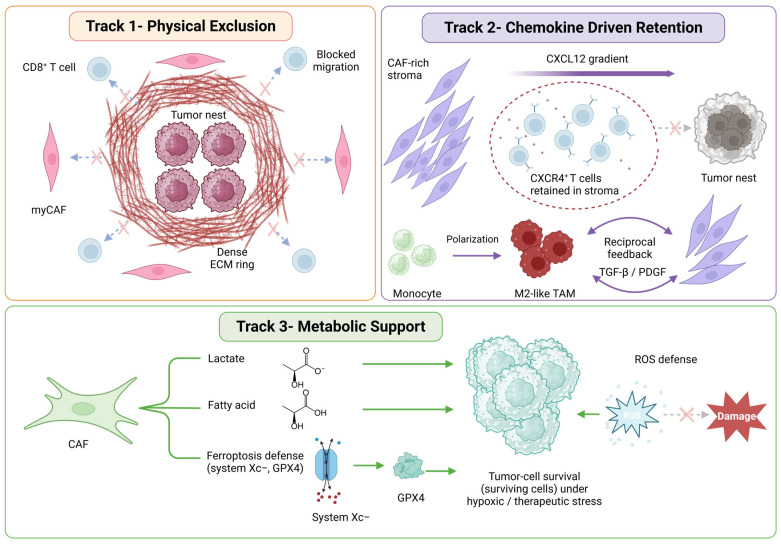
CAF-mediated immune exclusion and stromal adaptation in prostate cancer. CAF-rich stroma restricts antitumor immunity and supports tumor-cell survival through three non–mutually exclusive mechanisms. Track 1—Physical exclusion. Myofibroblast-like CAFs (myCAFs) deposit and remodel a collagen-rich extracellular matrix (ECM), forming a dense peritumoral ring that increases stromal stiffness, limits intratumoral transport, and physically restricts CD8^+^ T-cell entry into tumor nests (blocked-arrow symbols, ×). Track 2—Chemokine-driven retention. Inflammatory CAFs generate a CXCL12-rich stromal niche (additional chemokines, such as CCL2 and cytokines, such as IL-6 are not depicted) with CXCL12 concentration decreasing away from the CAF-rich stroma. CXCR4^+^ T cells migrate up this gradient and become retained within the niche (dashed boundary), preventing access to tumor nests. Recruited monocytes polarize toward an M2-like tumor-associated macrophage (TAM) phenotype; TAM- and CAF-derived TGF-β and PDGF then reciprocally reinforce this immunosuppressive stromal compartment. Track 3—Metabolic support. CAFs supply lactate, polyunsaturated fatty acids, and ferroptosis-defense factors—including the cystine/glutamate antiporter system Xc− and the lipid-peroxide–detoxifying enzyme GPX4—that sustain tumor-cell survival under hypoxic and therapeutic stress. ROS-mediated oxidative damage is neutralized by this CAF-supported antioxidant axis (ROS→×, Damage prevented). Created in BioRender. Kwon, W. (2026) https://BioRender.com/ft3vjoj accessed on 2 May 2026.

CAFs do not function in isolation. Their clinical influence often reflects coordination with other TME components, particularly immune cells and metabolic constraints. This coordination can promote immune exclusion within tumors, promote stress-adapted phenotypes, and establish niches that facilitate dissemination and metastatic growth (Figure 2). Although these effects are context-dependent, several recurrent mechanisms have been identified in PCa and in broader CAF biology [2,18].

### 4.1. Immune Exclusion and Immunosuppressive Circuits

Immune regulation is one of the most consistent translational themes in CAF biology. CAFs can affect immune-cell infiltration into tumors, their localization within the TME, and their phenotypes. Experimental evidence from PCa studies indicates that CAFs can recruit monocytes and induce M2 macrophage polarization, whereas M2 macrophages further enhance CAF reactivity. This reciprocal interaction supports tumor-cell motility and progression, thereby providing a direct mechanism through which fibroblasts shape a pro-tumorigenic TME [39].

CAF-TAM crosstalk represents a second immune-exclusion axis beyond direct CD8^+^ T-cell restriction. In PCa models, CAF-derived chemokine and cytokine signals (CCL2, CXCL12, CSF1, IL-6) recruit monocytes and promote M2-like macrophage polarization, whereas M2-like TAMs reciprocally reinforce CAF activation through TGF-β and PDGF, forming a feed-forward stromal-myeloid loop that increases tumor-cell motility [39,70]. Single-cell ligand-receptor analyses in PCa implicate FAP^+^ fibroblast-SPP1^+^ macrophage neighborhoods as candidate stromal-myeloid niches contributing to immune exclusion [74]. A FAP-high lesion with CD163/CD206-enriched TAMs should therefore be interpreted differently from FAP-high stroma with T-cell-proximal apCAF-like niches. These data support testing dual-compartment strategies rather than assuming that CAF-only or TAM-only targeting will be sufficient.

Chemokine signaling is central to this process. The CXCL12/CXCR4 axis has been highlighted as a major mediator of tumor-microenvironment communication, with implications for immune-cell trafficking and stromal organization [70]. In CAF-rich microenvironments, these chemokine gradients may facilitate immune exclusion by directing immune cells away from tumor nests or augmenting myeloid subsets that inhibit effector T-cell function [2,18]. Although immune phenotypes in PCa are complex, these data show that CAF activity can shape the immune landscape in manners relevant to response and resistance. Therefore, the immunomodulatory roles of CAFs are not uniform. Although inflammatory CAF (iCAF) and myofibroblastic CAF (myCAF) programs broadly sustain immune exclusion, as exemplified by iron-loaded CAF-driven immunosuppression, apCAF-like states may have divergent effects on T-cell localization and function depending on costimulatory-marker expression and the local CD4/CD8/Treg context, primarily inferred from non-prostate tumor models [29,41]. This context dependence has direct therapeutic implications: broad stromal depletion may eliminate immunostimulatory CAF subpopulations, potentially accelerating immune escape rather than enhancing antitumor immunity [38,46]. Systemic inflammatory states have also been linked to distinct immune landscapes in PCa, supporting integration of tissue and systemic immune context [75].

Beyond cytokine-mediated suppression, iron-loaded CAF programs in PCa tissue link immune modulation to metabolic specialization of fibroblast states (described in detail in Section 4.2) [10]. This suggests that single-pathway inhibition may be insufficient unless the broader state program is co-targeted [2,10]. Therefore, CAF state diversity should inform immune checkpoint trial design in PCa. Patient selection based on stromal immune phenotype, rather than tumor mutational burden alone, is required to identify patients who may benefit from immunotherapy combinations [2,18,19,76].

### 4.2. Metabolic Reprogramming and Oxidative Stress Programs

Metabolic crosstalk between CAFs and tumor cells can enhance aggressive phenotypes. CAFs can alter nutrient availability, supply metabolic intermediates, and reshape redox balance, thereby helping tumor cells manage stress induced by hypoxia, detachment, and therapy [34]. Altered amino acid availability and broader metabolic changes in the prostate TME may further influence tumor fitness and immune function [77]. This concept is particularly relevant in PCa, where metabolic plasticity is closely linked to progression and therapeutic resistance [78]. Stromal signals can also support tumor adaptation to external pressures [34]. In addition, stromal epigenetic alterations have been shown to drive metabolic rewiring and lineage-like reprogramming of PCa cells, supporting a causal role for the stroma in tumor adaptation [47].

Hypoxia amplifies these processes. Reviews of hypoxia-induced signaling in PCa emphasize that hypoxia can promote aggressive phenotypes and that exosome-mediated communication constitutes one mechanism through which hypoxic tumor and stromal cells exchange adaptive signals [65]. Hypoxia also affects stromal remodeling by promoting ECM deposition and altering vascular function. This interaction can further exacerbate oxygen and nutrient gradients, creating feedback loops that support stress-tolerant states [18,65].

The observation of iron-loaded CAFs adds another dimension to this metabolic framework. Mechanistically, ferroptosis defense programs, including Zeb1-linked control of system Xc-, may help sustain iron-loaded CAF states and their immunoregulatory effects [48]. Iron handling can influence oxidative stress, lipid peroxidation, and immune-cell function, and iron-loaded CAF programming has been linked to immunosuppressive phenotypes in PCa tissues [10]. Although the field has yet to define which metabolic CAF programs predominate across patient subsets, these findings suggest that CAF metabolic programs, including iron, redox, and lipid axes, should be integrated into the mechanistic rationale for combination strategies, with metabolic state profiling required to identify patients harboring therapeutically actionable stromal vulnerabilities [10,34].

Within the lipid axis, dependence on lipid metabolism for androgen synthesis, membrane biogenesis, and energy storage is a defining hallmark of PCa and a key basis for CAF-tumor metabolic crosstalk [79]. CAFs can supply lipid intermediates, including fatty acids and lysophospholipids, which fuel de novo lipogenesis in tumor cells under nutrient stress [80]. Conversely, tumor-derived signals may reprogram CAF lipid handling and promote a lipid-laden stromal phenotype that supports tumor-cell survival and immune evasion [81,82]. Cholesterol trafficking between CAFs and tumor cells has been implicated in sustaining AR signaling in CRPC, linking stromal lipid metabolism to treatment resistance [83,84]. These observations complement the iron-loading data and suggest that CAF metabolic states in PCa are multidimensional, encompassing redox, iron, and lipid axes, each potentially representing a distinct therapeutic vulnerability [81].

### 4.3. Dissemination and Bone Metastatic Microenvironment

CAF-related processes also intersect with dissemination and metastatic colonization. Metastasis requires not only the tumor-cell dissemination but also a supportive niche at a distant site. The bone microenvironment is particularly important in PCa. Reviews of bone metastasis biology emphasize that host microenvironmental components contribute to tumor seeding, survival, and outgrowth and that targeting the microenvironment is a rational strategy to improve outcomes [85].

The bone niche contains abundant stromal cells, ECM, and signaling molecules that facilitate dormancy and subsequent reactivation. Reviews emphasizing the microenvironment have elucidated how interactions among tumor cells, osteoblasts, osteoclasts, immune cells, and stromal constituents create feedback mechanisms that promote skeletal pathology [85]. Within this landscape, fibroblast-lineage stromal programs can remodel the ECM, shape cytokine and growth-factor availability, and coordinate immune suppression, thereby facilitating colonization and persistence [2,18].

A major translational implication is that metastatic niches may be shaped by systemic and treatment-related signals, not only by local tumor biology. Therapy-driven remodeling of stromal states has been demonstrated in PCa, and similar state shifts at metastatic sites may influence response and relapse patterns [9]. Therefore, site-specific and treatment-induced CAF state shifts should be longitudinally monitored in metastatic disease. Static, single-time point stromal assessments are insufficient to capture the dynamic stromal adaptation that drives relapse at metastatic sites [9,18].

In summary, CAFs promote tumor progression by orchestrating immune exclusion, metabolic adaptation, and niche formation. These mechanisms explain why CAF-rich ecosystems may resist tumor-directed therapies and why stromal biomarkers could enhance risk stratification. Furthermore, they underpin translational strategies involving the assessment of fibroblast activation across various compartments,.

## 5. Translational Assessment of CAF Activity

Progress in CAF biology is clinically useful only if CAF activity can be quantified using reliable, scalable methods associated with pertinent outcomes. Circulating growth and angiogenic factors, such as HGF and VEGF, can be measured in PCa cohorts and may change with interventions, although they are not CAF-specific readouts [86]. In PCa, this measurement challenge comprises three distinct layers. First, CAFs exhibit heterogeneity and are spatially organized, rendering a single marker insufficient to capture the entire biological complexity [19,22,35,49]. Second, CAF programs may evolve with therapy, making longitudinal tracking essential [9,50]. Third, different clinical questions require different tools, including risk stratification at diagnosis, monitoring during systemic therapy, and selection of patients for microenvironment-targeted approaches [18,42,87,88]. Below, we summarize translational readouts across tissue pathology, molecular signatures, liquid biopsy approaches, and FAP-targeted imaging.

### 5.1. Histopathology-Based Biomarkers

Histopathology remains the most accessible window into stromal biology as it is integrated into routine clinical practice. Reactive stromal grade provides a tissue-level assessment of fibroblast activation and matrix remodeling, and multiple studies support its association with clinically significant outcomes. Quantification of the stromogenic carcinoma area in prostatectomy specimens has identified patients at increased risk of PCa-specific mortality [16]. Additionally, population-based studies have demonstrated that RSG assessed on diagnostic needle biopsy has prognostic significance by correlating stromal status with long-term mortality risk early in clinical progression [7].

Reactive stroma also acquires clinical significance when combined with other adverse features, indicating microenvironmental invasion. Clinical pathology studies have examined reactive stroma together with perineural invasion and tumor grade, documenting associations with PCa-specific mortality [7]. Another study demonstrated that integrating lymphovascular invasion with RSG improved prediction of PCa mortality, consistent with the concept that activated stroma and vascular invasion collectively constitute a high-risk ecosystem [6]. Recent work has further incorporated reactive stromal assessment with glandular and acinar morphology, highlighting that stromal remodeling and epithelial architecture may collectively inform progression risk [37].

Beyond morphology, tissue-based detection of FAP is increasingly recognized as a clinically relevant stromal marker. Immunohistochemistry and correlative imaging indicate that intratumoral FAP distribution is quantifiable and potentially clinically meaningful [13]. Notably, stromal FAP expression is associated with MRI visibility and patient prognosis, underscoring the influence of stromal biology on imaging phenotypes and clinical outcomes [53]. Two priorities emerge: multicenter standardization of reactive stromal grading protocols and prospective validation of FAP IHC as a candidate stromal enrichment biomarker for stromal-targeting trials. Both are feasible within existing clinical infrastructure and warrant prospective validation [35,42].

### 5.2. Transcriptomic and Spatial Signatures

Molecular profiling enables a shift from describing stromal activation to defining state-resolved biological processes. Across tumor types, the gene expression patterns of CAFs exhibit shared and context-specific features, supporting the concept that fibroblast programs can be represented as modular signatures rather than isolated markers [89]. In PCa, integrated single-cell and bulk RNA sequencing approaches have been employed to characterize CAF heterogeneity and develop prognostic signatures, suggesting that fibroblast-state composition can be linked to patient outcomes through transcriptomic analyses [20].

Several recent datasets reinforce the translational relevance of state-based signatures in PCa. Single-cell analyses of prostate carcinogenesis describe fibroblast heterogeneity as an early and dynamic feature, supporting the hypothesis that stromal programs may contribute to risk stratification beyond the tumor epithelium [90]. Multi-omic single-cell integration further underscores that PCa heterogeneity is multi-compartmental, supporting combined epithelial-stromal signatures rather than tumor-only models [44]. A study describing distinct mesenchymal cell states as mediators of PCa progression provides a state-level framework directly relevant to biomarker development by linking mesenchymal states to functional roles in disease [49]. Spatial and integrated single-cell methodologies contribute significantly by delineating the localization of stromal programs and their associations with various TMEs and progression signatures [22].

Prostate-focused reviews have synthesized these approaches and highlighted biomarkers and therapeutic targets linked to CAFs, emphasizing the need for cross-platform standardization [35,87,91]. From a translational perspective, the immediate objective is not to catalog every fibroblast cluster. The field should prioritize clinical-grade validation of a consensus stromal signature panel that is cohort-robust, specimen-compatible, and independently prognostic, enabling future evaluation as a biomarker-driven enrollment criterion in interventional trials [20,22,35,49].

### 5.3. Circulating CAFs and Stromal Phenotypes in Blood

Liquid biopsy strategies offer important advantages as they enable repeated sampling and may detect dynamic alterations in the microenvironment throughout therapy. Early PCa studies documented circulating fibroblast-like cells in men with metastatic disease, supporting the feasibility of identifying stromal-like phenotypes in peripheral blood [92]. More recently, functional and heterogeneous cCAFs have been identified in mHSPC (also referred to as castration-naïve), indicating that circulating stromal states can be measured and may have biological significance rather than representing rare artifacts [11].

The circulating phase imposes biological constraints not captured by tissue CAF markers alone. Once released into blood, CAF-like cells encounter shear stress, anoikis pressure, platelet/leukocyte interactions, and short residence times. Experimental systems show that heterotypic clustering of cCAFs with circulating tumor cells increases shear resistance and reduces apoptosis during transit [93,94], supporting the hypothesis that some cCAF-containing clusters may contribute to metastatic transit in patients. Their translational relevance may therefore depend less on enumeration than on whether they form heterotypic clusters with CTCs, provide mechanical shielding under shear, or carry state-specific stromal programs from metastatic niches. Future cCAF reports should include capture method, viability, cluster status, platelet/leukocyte association, and concordance with tissue-defined CAF states.

Circulating CAFs have two complementary roles. First, they may provide a pharmacodynamic readout of stromal activation during systemic therapy, which is relevant as AR-targeted therapy can remodel stromal states and promote resistance-supporting programs [9,50]. Second, they may assist in monitoring metastatic progression and clarify the biology of dissemination, particularly when stromal cells interact with circulating tumor cells. Experimental studies indicate that CAFs can enhance the shear resistance of circulating tumor cells, supporting a mechanism by which stromal cells facilitate survival during metastatic progression [93]. Consistent with clinical interest in the circulation phase, blood-based strategies have been explored to directly target circulating tumor cells, demonstrating that dissemination can be studied as a biologically targetable compartment in hypothesis-generating settings [95]. Evidence from breast cancer and PCa indicates that cCAFs can be identified using heterogeneous marker combinations similar to those used for tissue CAFs [11,96], whereas recent automated liquid biopsy work further supports the feasibility of cCAF detection in breast cancer [97]. Additional evidence supports the plausibility of heterotypic clustering between circulating tumor cells and cCAFs, a phenomenon that may facilitate metastasis [94]. In vivo studies also show that cCAF detection can reflect tumor stroma and treatment effects in model systems [98]. Circulating CAF detection has been reported in other epithelial malignancies, helping define technical approaches and potential confounders [99].

Clinically, cCAF readouts may be most valuable in two contexts. First, they could enable longitudinal monitoring of stromal activation throughout systemic therapy, which is relevant because PCa treatment can modify CAF states and potentially enhance programs that support resistance [9,50]. Second, they may help identify dissemination phenotypes in advanced disease, particularly when integrated with other liquid biopsy layers [93,94]. However, their translational application is contingent on technical rigor. Circulating stromal-like cells are scarce, and their definition depends heavily on enrichment methodologies and marker panels [11,99]. Harmonization is therefore essential, including standardized reporting of capture techniques, marker definitions, validation procedures, reproducibility metrics, and explicit connections between a circulating phenotype and tissue-defined CAF program. Their use as co-primary trial endpoints therefore remains premature [11,35].

Despite their potential, cCAF assays face critical technical barriers [100]. First, no standardized enrichment protocol exists, and epithelial cell adhesion molecule (EpCAM)-negative depletion strategies may co-deplete stromal-like cells, increasing false-negative rates [100]. Second, cCAFs lack a lineage-defining marker: FAP, αSMA, and PDGFRβ are also expressed by non-fibroblast blood populations, including activated platelets and mesenchymal stem cells, making specificity a persistent concern [101]. Third, functional heterogeneity among cCAFs, as demonstrated in mHSPC, implies that bulk enumeration without state characterization may obscure clinically relevant subpopulations [11]. Fourth, the circulating half-life, shedding dynamics, and extent to which cCAFs reflect primary versus metastatic stroma remain undefined [102]. Until multisite validation establishes concordance between cCAF phenotypes and tissue-defined CAF programs, cCAF readouts should be considered hypothesis-generating rather than practice-informing biomarkers [100,102].

### 5.4. FAP-Targeted Imaging and Theranostics

Non-invasive mapping of fibroblast activation has advanced significantly with the development of FAP-targeted PET imaging. In metastatic PCa, imaging of FAP expression has been shown to improve PET-based diagnosis, supporting activated stroma as a valuable imaging target alongside tumor-centric tracers [59]. Case reports and small series highlight a clinically relevant application: FAPI PET may be positive in mHSPC characterized by prostate-specific membrane antigen (PSMA)-negative and fluorodeoxyglucose (FDG)-positive disease, indicating potential utility in phenotypes with conventional tracer discordance [60]. FAP-targeted imaging has also been reported in treatment-naive PCa patients with low PSMA expression, thereby enabling supplementary detection across disease stages [58].

Multiple studies underscore its relevance in advanced disease. Elevated FAP expression in CRPC underpins the rationale for FAP-targeted theranostics in this context [12]. Comparative imaging studies evaluate FAP-targeted PET alongside PSMA and FDG, reflecting efforts to determine when FAP provides information beyond existing imaging standards [61]. Integration of immunohistochemistry and imaging offers preliminary insights into the relationship between PSMA and FAP distribution in PCa tissues, which is essential for interpreting discordant tracer uptake and developing dual-target strategies [13]. A systematic review of FAP inhibitor PET/CT in genitourinary cancers further summarized its diagnostic performance and helped delineate the limitations and knowledge gaps across indications [62]. Recent multi-tracer comparisons in high-risk PCa further clarify how FAPI PET performs relative to PSMA PET and FDG PET in the same patient cohort [63].

The translational pathway inherently progresses from imaging to theranostics and then to dual-target concepts. Early discussions and preliminary clinical reports have outlined potential applications of FAP inhibitors in PCa theranostics [54,55]. Dual-target strategies that integrate PSMA and FAP aim to bridge tumor-cell and stromal targeting, supported by preclinical dual-target probes and comprehensive reviews describing the rationale and design considerations [51,103]. Clinically, FAPI-PET has been described as a complementary modality in PSMA-negative cases, supporting combined strategies for managing heterogeneous disease [57].

**Figure 3:**
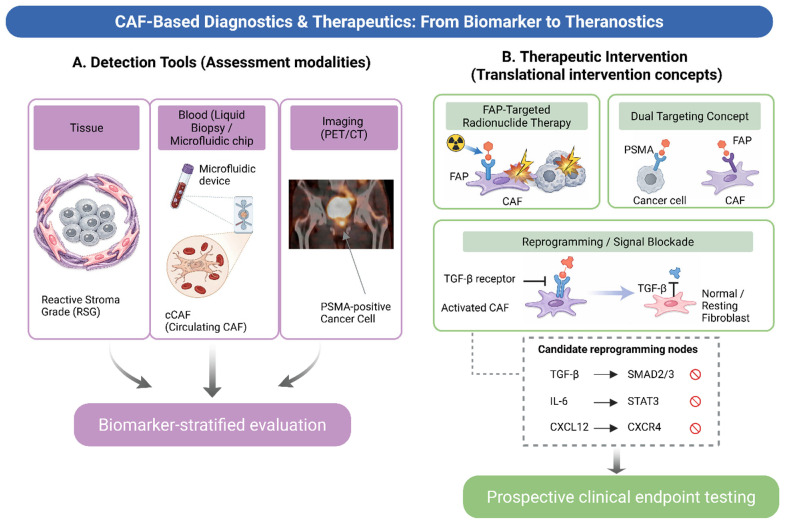
CAF-based diagnostic and therapeutic framework in prostate cancer: from biomarker assessment to stromal theranostics. (**A**) Detection tools. CAF/stromal assessment is based on three complementary modalities: (i) tissue-based scoring of the Reactive Stromal Grade (RSG) on histology; (ii) blood-based capture of circulating CAFs (cCAFs) via microfluidic devices for liquid biopsy, and (iii) imaging-based PET/CT—PSMA-PET is illustrated as the current standard-of-care imaging modality for prostate cancer, and analogous CAF-targeted PET strategies (e.g., FAPI-PET) may be combined with PSMA-PET to assess tumor and stromal compartments. Together, these modalities support biomarker-guided trial design and evidence-tiered patient stratification. (**B**) Therapeutic intervention. Three mechanism-matched strategies are shown: (i) FAP-targeted radionuclide therapy that delivers cytotoxic radiation to FAP-expressing CAFs, (ii) dual targeting of PSMA-positive tumor cells and FAP-positive CAFs to address malignant and stromal compartments concurrently, and (iii) CAF reprogramming/signal blockade that reverts activated CAFs toward a normal/resting fibroblast phenotype via inhibition of TGF-β receptor signaling. Candidate reprogramming nodes (inset)—TGF-β/SMAD2/3, IL-6/STAT3, and CXCL12/CXCR4—represent pharmacologically blockable axes that link diagnostic CAF profiling to mechanism-matched therapeutic intervention, supporting prospective evaluation of clinical endpoints in biomarker-stratified trials. Created in BioRender. Kwon, W. (2026) https://BioRender.com/s2174f5.

Overall, FAP-targeted imaging provides a direct approach for assessing stromal activation in vivo and may inform patient selection in PSMA-discordant disease after prospective validation, while also supporting therapy-oriented hypothesis generation (Figure 3). Establishing its clinical value will require prospective trials in which FAPI PET avidity serves as an enrollment criterion, not merely a descriptive correlate, thereby enabling a rigorous assessment of whether stromal imaging translates into a survival benefit beyond its current diagnostic utility [42,62,103].

## 6. Targeting CAF Programs: Therapeutic Strategies and Trial Design

Although CAF biology presents compelling intervention opportunities, it also introduces potential challenges. CAFs are heterogeneous, and their functions range from tumor-promoting to context-dependent. Accordingly, many stromal-targeting strategies focus on modulating CAF programs rather than depleting all fibroblasts [2,18]. In PCa, therapeutic interventions may alter the stromal environment and increase CAF states that support resistance, providing a rationale for combination strategies that account for stromal adaptation [9,50]. Selective depletion remains rational when targeting is state-specific; FAP-targeted chimeric antigen receptor T cell (CAR-T), immunotoxins, and bispecific constructs aim to eliminate immunosuppressive FAP-high stroma while sparing quiescent fibroblasts, thereby enhancing cytotoxic T-cell infiltration without broadly disrupting stromal architecture [104,105]. Conversely, strong counterevidence comes from preclinical αSMA-CAF depletion models, most extensively characterized in pancreatic cancer, in which ablation of myofibroblast-like CAFs paradoxically worsened hypoxia, expanded regulatory T cells, and accelerated rather than restrained disease. These data suggest that therapeutic outcome depends on the functional identity of the targeted subpopulation, not depletion per se [43,105]. Therefore, selecting the appropriate strategy depends on which CAF program predominates, which frequently demands combination approaches rather than single-agent stromal targeting. Reviews of emerging therapeutic strategies for PCa reinforce this need for rational combinations [106,107].

CAF state diversity has direct therapeutic implications: different stromal programs require different interventions, and a single stromal strategy applied to an unselected population is unlikely to succeed [107,108]. We therefore propose a “Stromal Precision Medicine” framework built on three linked steps: (1) classify the dominant CAF program using tissue-based readouts (e.g., RSG, FAP IHC, or spatial transcriptomics) or blood-based readouts (e.g., cCAF phenotype) [45,109]; (2) match the identified program to a mechanism-aligned intervention (Table 3) [107]; and (3) monitor on-target stromal modulation using pharmacodynamic biomarkers before assessing clinical endpoints [55,107]. This framework parallels tumor-centric precision oncology paradigm and is supported by emerging biological evidence relevant to early-phase trial design [107,108,110].

This caution is reinforced by translational failures in stromal-targeting programs. Pegvorhyaluronidase alfa (HALO-301) showed no survival benefit in hyaluronan-high pancreatic cancer despite biological activity [67]; broad TGF-β inhibition has been limited by cardiotoxicity-driven therapeutic windows [111]; and pancreatic Hedgehog-pathway stromal depletion (IPI-926) accelerated rather than restrained progression [112]. These outcomes converge on a recurring lesson: stromal targeting without state-resolved patient selection has repeatedly underperformed despite strong biological plausibility, supporting the decision-rule framework shown in Table 3.

### 6.1. Depletion Versus Reprogramming: A State-Matched Decision Rule

A practical decision rule is needed to avoid presenting depletion and reprogramming as unresolved competing narratives. Selective depletion is rational only when a targetable, spatially dominant, tumor-promoting CAF state is demonstrated, such as high FAP uptake/IHC in immune-excluded, TAM-rich lesions with low intratumoral CD8^+^ T-cell access, and when on-target stromal monitoring is available. Reprogramming or normalization is safer when CAF states are mixed, αSMA/myCAF-dominant, perivascular or bone-niche associated, or spatially linked to apCAF/T-cell neighborhoods. ECM-directed approaches should normalize stiffness, transport, and immune access rather than indiscriminately dissolve matrix barriers. Table 3 operationalizes this decision rule by linking dominant CAF context to preferred strategy, pitfalls to avoid, biomarker-endpoint, and evidence tier; full intervention-level detail, including drug classes, NCT trial identifiers, and delivery platforms, is provided in Supplementary Table S2 [5,6,7,9,11,12,15,16,18,30,35,42,50,51,53,57,59,61,63,70,71,74,88,103,113,114,115,116,117].

The central therapeutic question is whether CAFs should be eliminated or whether their activity should be modulated. Comprehensive reviews of stromal biology highlight substantial risks from non-selective stromal depletion, because fibroblasts have multiple homeostatic and context-dependent functions. Additionally, removal of the stromal compartment may induce compensatory signaling pathways or favor more aggressive phenotypes [2,18,118]. Prostate-specific research reinforces this caution: stromal programs can substantially influence epithelial behavior, and stromal modification may redirect tumor phenotypes rather than suppress growth [3,4,69].

Consequently, many contemporary approaches prioritize reprogramming and functional blockade over cellular depletion. Reviews focused on PCa, CAFs, and resistance suggest that targeting pathways that maintain pro-tumor CAF states may be more tractable than extensive ablation [42,91]. This framing also aligns with evidence that therapy itself can reprogram stromal states and promote castration resistance, implying that the stroma is plastic and potentially reprogrammable [9,50]. Candidate reprogramming strategies include vitamin D receptor agonists, which have been shown to normalize pancreatic stellate cells toward a quiescent phenotype [119] and may represent a translatable stromal-normalization approach in PCa [107]. All-trans retinoic acid and selective TGF-β pathway inhibition are additional mechanistic options warranting prospective evaluation in stromal-high PCa cohorts [9,107].

### 6.2. Targeting Upstream Activation Pathways

CAF programs are supported by a restricted set of signaling pathways that can be pharmacologically modulated. TGF-β signaling is a frequently observed pathway strongly associated with fibroblast activation, matrix remodeling, and immunosuppressive microenvironments across cancers [2,18]. In PCa, targeting a microenvironmental axis influenced by *HIC1* and TGF-β has been reported to inhibit disease progression, thereby supporting the hypothesis that stromal state can be therapeutically modulated through specific upstream regulators [113].

Chemokine signaling provides another tractable axis. Reviews of tumor-stroma communication in PCa emphasize chemokines as pivotal organizing signals within the TME [88]. Prostate-focused studies have also identified the CXCL12/CXCR4 axis as a mechanism regulating the spatial predominance of regulatory T cells over CD8^+^ T cells through IL-2 sequestration, positioning this axis as a dual therapeutic target [114]. These findings support a pragmatic strategy: blocking the stromal-to-immune circuits that sustain immune exclusion, particularly when CAF states are associated with immunosuppressive microenvironments [42,74,114].

A third category involves tumor-stroma signaling interfaces linked to the androgen pathway. AR-targeted therapy may induce stromal reprogramming and promote castration resistance through specific ligand-receptor circuits, providing a compelling rationale for integrating AR-targeted therapy with strategies that disrupt downstream stromal support mechanisms [9]. Research on the AR-filamin A complex also highlights microenvironment-associated signaling interfaces as viable therapeutic targets in PCa [52]. Collectively, these data suggest that CAF targeting warrants prospective testing within context-matched treatment strategies rather than being regarded as a static adjunct [9,50,88]. Figure 3 (inset) summarizes three principal candidate reprogramming nodes—TGF-β/SMAD, IL-6/STAT3, and CXCL12/CXCR4—as pharmacologically blockable axes linking diagnostic CAF profiling to mechanism-matched therapeutic intervention.

### 6.3. Targeting Extracellular Matrix Remodeling

Because matrix remodeling is a core CAF output, ECM-directed strategies—targeting deposition, crosslinking, or mechanotransduction—represent rational interventions to attenuate pro-invasive and stress-tolerant programs [2,5,18]. Pathological and imaging readouts of fibroblast activation can help identify patients most likely to benefit from matrix-normalizing combinations [35,42].

### 6.4. FAP-Targeted Therapies and Rational Combinations

FAP is considered advantageous because of its association with activated fibroblasts and its amenability to in vivo measurement. Elevated FAP expression in CRPC supports FAP-targeted theranostic approaches in advanced disease [12]. Early clinical discussions and preliminary reports have outlined potential roles for FAP inhibitors in PCa theranostics, thereby reinforcing the transition from imaging to therapeutic intervention [54,56].

An important limitation is that FAP targeting alone may not sufficiently address tumor-cell heterogeneity. This limitation underscores the need for dual-target strategies that integrate tumor- and stroma-directed targeting. Preclinical studies of dual FAP/PSMA probes support the feasibility of noninvasive dual targeting in PCa models [51], and a recent theranostic review describes how dual targeting of PSMA and FAP can integrate tumor detection with stromal biology to strengthen precision strategies [103]. In practice, the most compelling clinical scenarios involve tracer discordance or heterogeneous target expression, where targeting both compartments may mitigate blind spots [57,103,115].

Rational combinations should also account for therapy-induced stromal editing. Because AR-targeted therapy can reprogram CAF states toward resistance-supporting pathways, CAF-directed interventions may be most effective when strategically timed to prevent or mitigate this shift rather than after resistance is fully established [9,50]. Similarly, system-level evaluations of microenvironment targeting in PCa emphasize that combination strategies will likely be necessary to translate stromal insights into durable benefits [88,117].

Nanoparticle-based approaches offer alternative methods for targeting tumor and stromal tissues. A comprehensive review elucidated strategies for directing nanoparticles to prostate tumors and their stromal components, supporting delivery methods that may enhance penetration and specificity in diseases characterized by abundant stroma [116]. These delivery platforms may be particularly advantageous when a potent therapeutic payload requires exposure within specific microenvironments.

### 6.5. Biomarker-Guided Trial Concepts and Endpoints

Considering the heterogeneity of CAF, clinical testing should be guided using specific biomarkers. Reviews of prostate CAF biology emphasize the importance of aligning interventions with the predominant CAF program within a specific patient group and utilizing quantifiable indicators of stromal modulation as pharmacodynamic endpoints. Potential trial designs include enrichment based on tissue stromal features, such as RSG and FAP expression [7,12,53], and response monitoring using tissue, blood, or imaging readouts to confirm on-target microenvironmental changes [11,54,59]. Combination trials pairing androgen pathway suppression with agents that have microenvironment-relevant activity have been explored in PCa, supporting the feasibility of rational combination designs [120] (see Supplementary Table S3 for a critical translational appraisal of selected trials).

## 7. Conclusions

CAFs are not passive architectural elements of the prostate TME; they are dynamic, state-switching effectors whose composition at any disease stage reflects the cumulative history of tumor evolution, therapeutic exposure, and microenvironmental crosstalk. The central argument of this review is that fibroblast heterogeneity is not merely a biological curiosity, but a mechanistic determinant of progression and resistance that requires state-resolved characterization before rational intervention can be pursued.

Clinically, this synthesis supports gradual incorporation of stromal biology into trial design and patient management rather than immediate changes in practice. Reactive stromal grade and FAP immunohistochemistry warrant prospective evaluation as candidate enrichment or stratification variables in trials targeting the TME, rather than collection only as exploratory correlates after enrollment. FAP-targeted PET imaging is supported by emerging evidence as a complementary modality in PSMA-discordant or tracer-heterogeneous disease and may inform patient selection for theranostic strategies after prospective validation. In patients receiving AR-targeted therapy, longitudinal stromal monitoring, primarily tissue-based and complemented by cCAF assays as exploratory adjuncts, may help detect resistance-supporting stromal reprogramming before it becomes entrenched. The stromal precision medicine framework proposed here links dominant CAF program classification to mechanism-aligned intervention and pharmacodynamic endpoint selection, providing an operational structure for this gradual translation.

Current recommendations are limited by the absence of prospectively validated, cohort-robust stromal signature panels; the lack of standardized cCAF enrichment and phenotyping protocols; and the near-complete absence of stromal pharmacodynamic endpoints in completed CAF-relevant trials, which restricts mechanistic interpretation of existing efficacy data.

Three research priorities follow from this synthesis. First, prospective multi-institutional validation is warranted for a consensus tissue-based stromal signature panel that is specimen-compatible and independently prognostic across disease stages. Second, harmonization studies should establish concordance between cCAF phenotypes and tissue-defined CAF programs before liquid biopsy deployment. Third, biomarker-stratified window-of-opportunity trials should test whether stromal program modulation, confirmed by on-target pharmacodynamic endpoints, translates into measurable clinical benefit in defined CAF-high patient subgroups.

## Figures and Tables

**Table 1:** Operational marker panels and their blind spots in CAF identification.

Readout	Likely Enriched Program	Main Blind Spot
ACTA2/αSMA + COL1A1/COL3A1	myCAF/ECM-contractile	Misses iCAF, apCAF-like, metabolic CAFs
FAP + PDGFRβ + COL1A1	Activated/FAP-high stroma	Does not equal “all CAFs”; may include perivascular/MSC-like cells
CXCL12, IL6, CCL2, LIF	iCAF/secretory-immune	Requires lineage exclusion and spatial context
HLA-DRA, CD74, MHC-II; low ACTA2	apCAF-like	Function depends on CD4/CD8/Treg proximity and costimulation
Iron/redox/lipid markers	Metabolic CAF candidates	No standardized clinical panel
Pre/post-therapy ligand–receptor shifts	Therapy-imprinted CAFs	Requires paired sampling; not captured by baseline IHC

Representative marker-panel assignments and limitations are based on CAF biomarker literature and PCa single-cell/translational profiling studies [2,9,19,20,21,22,23]. FAP-high, αSMA-high, and PDGFRβ-positive compartments should be interpreted as overlapping operational readouts, not interchangeable definitions of the CAF population.

**Table 2:** Operational summary of CAF programs in prostate cancer.

CAF Program	Minimal Marker/Readout	Mechanism (One-Line)	Evidence Maturity in PCa
myCAF/ECM-contractile	ACTA2/αSMA + COL1A1/COL3A1	Matrix deposition, stiffness, transport barrier	Established
iCAF/secretory-inflammatory	CXCL12, IL6, CCL2, LIF	Paracrine tumor support, myeloid recruitment	Prostate-supported
apCAF-like	HLA-DRA, CD74, MHC-II (low ACTA2)	Context-dependent immune modulation in stromal niches	Cross-tumor inferred
Metabolic/immunoregulatory	Iron/redox/lipid markers	Metabolic-immune crosstalk; ferroptosis defense	Prostate-supported
Therapy-imprinted	Pre/post-therapy ligand–receptor shifts	Adaptive support of castration resistance	Prostate-supported
FAP-enriched activated stroma	FAP IHC/FAP-PET	Imaging-detectable fibroblast activation; theranostic target	Prostate-supported

Evidence tiers: Established (validated prognostic correlate with reproducible clinical data); Prostate-supported (PCa-specific translational evidence, validation ongoing); Cross-tumor inferred (extrapolated from non-PCa atlases, prostate-specific data limited); Exploratory (early evidence, hypothesis-generating only). This table is a streamlined operational summary; detailed evidence and citations are provided in Supplementary Table S1 [1,2,5,6,7,9,10,11,12,13,15,16,18,19,20,21,22,24,25,26,27,29,31,32,33,34,35,36,37,38,39,40,41,42,43,44,45,46,47,48,49,50,51,52,53,54,55,56,57,58,59,60,61,62,63].

**Table 3:** Decision framework for CAF-directed intervention in prostate cancer.

Dominant CAF Context	Preferred Strategy	What to Avoid	Biomarker/Endpoint	Evidence Tier
FAP-high, TAM-rich, CD8-excluded stroma	Selective FAP-directed targeting ± immune modulation	Broad fibroblast depletion	FAP-PET/IHC, intratumoral CD8 access, CD163/CD206 TAM density	Prostate-supported
αSMA/COL-rich myCAF-dominant stroma	ECM normalization/transport modulation	Aggressive matrix dissolution	RSG, collagen architecture, delivery surrogate	Prostate-supported
apCAF-like, T-cell-proximal niche	Preserve or reprogram	Indiscriminate depletion	MHC-II/CD74 + CD4/CD8/Treg co-registration	Cross-tumor inferred
AR-targeted-therapy-induced stromal shift	Combine AR-pathway inhibition with stromal modulation	Late-stage single-agent stromal targeting	Pre/post-treatment stromal signatures; cCAF (exploratory)	Prostate-supported
cCAF-positive blood phenotype	Exploratory monitoring only	Clinical decision-making based on cCAF alone	Capture method, cluster status, tissue concordance	Exploratory
Mixed/uncharacterized stroma	Defer stromal-targeting until classification	Empiric stromal therapy	Tissue + imaging stromal phenotyping	Exploratory

This author-derived framework links the dominant CAF context to a preferred mechanism-matched strategy, explicit pitfalls to avoid, pharmacodynamic biomarkers and endpoints, and evidence tier. Evidence tiers are defined in the Table 2 footnote; detailed supporting citations for the underlying CAF programs and candidate interventions are provided in Supplementary Tables S1 and S2.

## Data Availability

No new data were created or analyzed in this study. Data sharing is not applicable to this article.

## Supplementary Materials

The following supporting information can be downloaded at: https://www.mdpi.com/article/10.3390/cancers18101600/s1, Table S1. Detailed operational and translational characterization of CAF programs in prostate cancer (extended version of Table 2). Table S2. Actionable CAF programs, candidate interventions, disease settings, and suggested biomarkers for biomarker-guided trials in PCa. Table S3. Critical translational appraisal of selected CAF-targeted clinical and translational studies in prostate cancer and adjacent solid-tumor settings.

## Abbreviations

The following abbreviations are used in this manuscript:

ACTA2	actin alpha 2, smooth muscle
ADT	androgen deprivation therapy
apCAF	antigen-presenting cancer-associated fibroblast
AR	androgen receptor
CAF	cancer-associated fibroblast
CAR-T	chimeric antigen receptor T cell
cCAF	circulating cancer-associated fibroblast
CCL2	C-Cmotif chemokine ligand 2
CD8	cluster of differentiation 8
CD74	cluster of differentiation 74
COL1A1	collagen type I alpha 1
COL3A1	collagen type III alpha 1
CRPC	castration-resistant prostate cancer
CSF1	colony-stimulating factor 1
CXCL12	C-X-C motif chemokine ligand 12
CXCR4	C-X-C motif chemokine receptor 4
ECM	extracellular matrix
EMT	epithelial–mesenchymal transition
EpCAM	epithelial cell adhesion molecule
FAP	fibroblast activation protein
FAPI	fibroblast activation protein inhibitor
FDG	fluorodeoxyglucose
GPX4	glutathione peroxidase 4
HGF	hepatocyte growth factor
HLA-DRA	major histocompatibility complex class II DR alpha
iCAF	inflammatory cancer-associated fibroblast
IHC	immunohistochemistry
IL-2	interleukin-2
IL-6	interleukin-6
LIF	leukemia inhibitory factor
LVI	lymphovascular invasion
mCRPC	metastatic castration-resistant prostate cancer
MET	mesenchymal–epithelial transition factor
MHC	major histocompatibility complex
mHSPC	metastatic hormone-sensitive prostate cancer
MRI	magnetic resonance imaging
MSC	mesenchymal stromal cell
myCAF	myofibroblastic cancer-associated fibroblast
NCT	ClinicalTrials.gov identifier
PCa	prostate cancer
PD-L1	programmed death-ligand 1
PDGF	platelet-derived growth factor
PDGFRβ	platelet-derived growth factor receptor beta
PET	positron emission tomography
PET/CT	positron emission tomography/computed tomography
PSA	prostate-specific antigen
PSMA	prostate-specific membrane antigen
ROS	reactive oxygen species
RSG	reactive stromal grade
scRNA-seq	single-cell RNA sequencing
TAM	tumor-associated macrophage
TGF-β	transforming growth factor-beta
TME	tumor microenvironment
Treg	regulatory T cell
VEGF	vascular endothelial growth factor
VEGFR2	vascular endothelial growth factor receptor 2
αSMA	alpha-smooth muscle actin
