# Supplementary material for: Deciphering the Heterogeneity of Cancer-Associated Fibroblasts in Prostate Cancer: From Stromal Biology to Clinical Translation
Source: Cancers (Basel). 2026 May 14;18(10):1600. doi: 10.3390/cancers18101600 (PMC13204367; doi:10.3390/cancers18101600)
Supplement: Supplementary file 1 [file cancers-18-01600-s001.zip › cancers-4268695-supplementary.pdf]

cancers

MDPI

Supplementary Table S1.Detailed operational and translational characterization of CAF programs in prostate cancer (extended version of Table 2) [1,2,5–7,9–13,15,16,18–22,24–27,29,31–63].

CAF program	Representative features and markers	Key functions	Clinical relevance	Patient measurement
ECM remodeling and contractile programs	Collagen/ECM-rich, *ACTA2*/αSMA-high, myofibroblast-like states [2,19,35]	Matrix deposition, tissue stiffening, invasion tracks, and transport barriers [1,5,15,18,36]	Associated with adverse pathology and outcome in localized/high-risk disease; candidate phenotype for ECM-normalizing strategies [7,9,16,18,37]	Reactive stroma grading, stromal morphology, and multi-marker IHC panels [6,7,16,37]
Inflammatory and secretory programs	Cytokine- and chemokine-rich inflammatory CAF states described across tumor types [24,25,33,38]	Paracrine tumor support, immune-cell recruitment, and myeloid skewing [1,18,39,40]	Supports immune-context phenotyping and rationale for immunomodulatory combinations in CAF-rich tumors [39,41–43]	Bulk, single-cell, and spatial transcriptomic signatures; immune-neighborhood mapping [20–22,44,45]
Antigen-presenting programs (apCAF)	MHC class II-high, CD74+, typically low αSMA; spatially linked to immune cell-rich stromal niches [26,27,32]	Local antigen presentation and context-dependent immune modulation within stromal niches [26,29,31]	May define an immunoregulatory or immunostimulatory stromal minority; cautions against indiscriminate stromal depletion; prostate-specific predictive value remains unvalidated [29,31,46]	Spatial transcriptomics, scRNA-seq immune-neighborhood mapping, and MHC class II-oriented tissue profiling with immune co-registration [22,27,32]
Immunoregulatory and metabolic programs	Metabolically rewired CAF states, including iron-loaded immunosuppressive programs [10,34,47,48]	Immune suppression, redox adaptation, stress tolerance, and metabolic support of tumor growth [10,34,47]	Marks immunosuppressive microenvironments and supports metabolic-immune combination strategies [10,34,42]	State-resolved signatures with spatial context; functional markers where validated [10,22,49]
Therapy- or context-imprinted programs	Stromal states reshaped by treatment or disease context; resistance-supporting circuits [50,51]	Adaptive support of castration resistance and persistent microenvironmental remodeling [50–52]	Supports treatment-stratified biomarker development and earlier stromal combination approaches with AR-directed therapy [42,50,51]	Pre/post-treatment tissue profiling, longitudinal stromal signatures, and exploratory cCAF monitoring [11,50,51]
FAP-enriched activated stroma	FAP-positive activated stroma with imaging-detectable fibroblast activity [12,13,19,53]	Broad marker of stromal activation and candidate imaging/theranostic target [12,54–56]	Supports selection for FAP imaging and FAP-targeted theranostics, especially in PSMA-low or tracer-discordant disease [57–60]	IHC with imaging correlation; FAP/FAPI PET [59,61–63]

This supplementary table preserves the full citation density and detailed clinical-relevance and patient-measurement descriptions from the original manuscript. Table 2 provides a streamlined summary for rapid clinical reading. Abbreviations: *ACTA2*, actin alpha 2, smooth muscle; apCAF, antigen-presenting cancer-associated fibroblast; AR, androgen receptor; CAF, cancer-associated fibroblast; cCAF, circulating cancer-associated fibroblast; CD74, cluster of differentiation 74; ECM, extracellular matrix; FAP, fibroblast activation protein; FAPI, fibroblast activation protein inhibitor; IHC, immunohistochemistry; MHC, major histocompatibility complex; PCa, prostate cancer; PET, positron emission tomography; PSMA, prostate-specific membrane antigen; scRNA-seq, single-cell RNA sequencing; αSMA, alpha-smooth muscle actin.

 https://doi.org/10.3390/cancers18101600

Supplementary Table S2. Actionable CAF programs, candidate interventions, disease settings, and suggested biomarkers for biomarker-guided trials in PCa [5–7,9,11,12,15,16,18,30,35,42,50,51,53,57,59,61,63,70,71,74,88,103,113–117].

Target CAF program	Candidate intervention approaches	Supporting evidence	Most suitable disease setting	Suggested biomarkers and endpoints
CAF activation via TGF-β-linked circuits	Inhibit upstream CAF activation or downstream TGF-β-associated effectors	TGF-β-shaped stromal circuits promote PCa progression and taxane resistance [71,113]	Stromal-high mHSPC/mCRPC, especially resistance-prone or post-taxane disease	Stromal activation signatures; reactive stroma grading [6,7]; tissue or imaging evidence of on-target modulation [35,42]
Chemokine-driven immune exclusion	Disrupt CXCL12/CXCR4-related circuits; combine with immunotherapy where appropriate	CXCL12/CXCR4 signaling supports immune suppression and Treg dominance in PCa [30,70]; stromal macrophage crosstalk further supports immune-remodeling strategies [74]	Immune-excluded or myeloid-biased advanced disease	Spatial immune metrics; CAF-immune signatures; CD8/Treg balance; immune-remodeling endpoints [74,114]
AR-context stromal support during therapy	Combine AR-pathway inhibition with stromal modulation; block resistance-supporting stromal circuits	AR-targeted therapy reprograms stroma and promotes castration resistance [9]; persistent stromal evolution is observed after this therapy [50]	mHSPC and early CRPC during AR-targeted therapy	Pre/post-treatment stromal signatures; longitudinal tissue or blood monitoring, including exploratory cCAF assessment [9,11,50]; time-to-resistance endpoints
ECM remodeling and transport barriers	Target ECM production, crosslinking, or mechanotransduction; combine to improve penetration or response	Reactive stroma and ECM remodeling contribute to progression and treatment limitation [5,15,18]	Localized high-risk disease, bulky tumors, or stromal-rich lesions	Reactive stroma grading and stromogenic area [6,7,16]; imaging surrogates where relevant [18,59]; response-depth or delivery-related endpoints
FAP-targeted and theranostics	Use FAPI-based imaging and theranostic strategies; deliver payloads to activated stroma	High FAP expression in CRPC and early theranostic studies support this approach [12,53,115]	mCRPC with high FAP uptake, especially PSMA-low or tracer-discordant disease	FAP/FAPI PET for selection and monitoring [59,61,63]; lesion-level response and progression endpoints
Dual tumor-stroma targeting	Apply dual PSMA/FAP imaging or therapeutic strategies	Preclinical dual-target feasibility and translational rationale have been reported [51,103]	Metastatic heterogeneous disease with mixed or discordant tracer uptake	Multitracer imaging frameworks; lesion-level concordance and whole-patient progression endpoints [57,103]

https://doi.org/10.3390/cancers18101600

Target CAF program	Candidate intervention approaches	Supporting evidence	Most suitable disease setting	Suggested biomarkers and endpoints
Delivery platforms to reach stromal-rich tumors	Use nanoparticle-based tumor/stroma targeting and microenvironment-directed payload delivery	Delivery-based stromal targeting strategies have been proposed in PCa [88,116]	Localized or locally advanced stromal-rich disease with suspected penetration constraint	Pathology- or imaging-defined stromal enrichment; tissue or blood pharmacodynamic readouts [35,116]
Trial design for microenvironment targeting	Match combinations, enrichment, and endpoints to the dominant measurable CAF program	Reviews support mechanism-matched combinations and biomarker-guided trial design [88,117]	Applicable across disease states when a dominant CAF program is measurable	Serial tissue, blood, cCAF, or imaging biomarkers, including FAP PET where relevant [11,35]

Abbreviations: ADT, androgen deprivation therapy; AR, androgen receptor; CAF, cancer-associated fibroblast; cCAF, circulating cancer-associated fibroblast; CD8, cluster of differentiation 8; CRPC, castration-resistant prostate cancer; CXCL12, C-X-C motif chemokine ligand 12; CXCR4, C-X-C motif chemokine receptor 4; ECM, extracellular matrix; FAP, fibroblast activation protein; FAPI, fibroblast activation protein inhibitor; mCRPC, metastatic castration-resistant prostate cancer; mHSPC, metastatic hormone-sensitive prostate cancer; PCa, prostate cancer; PET, positron emission tomography; PSMA, prostate-specific membrane antigen; RSG, reactive stromal grade; TGF-β, transforming growth factor-beta; Treg, regulatory T cell.

Supplementary Table S3. Critical translational appraisal of selected CAF-targeted clinical and translational studies in prostate cancer and adjacent solid-tumor settings.

Trial/Agent	Target	Design	Setting	Representative NCT/Trial Identifier	Translational Considerations
Bintrafusp alfa	TGF-β + PD-L1	Phase I	Advanced solid tumors	NCT02517398	Strong dual-pathway rationale, but systemic TGF-β blockade may narrow the therapeutic window; no stromal biomarker enrichment was incorporated.
Cabozantinib + ADT	MET/VEGFR2 + AR	Phase II	mHSPC	NCT01630590	Positive efficacy signal with plausible stromal relevance through MET-linked paracrine signaling; absence of pre-specified stromal endpoints limits mechanistic attribution.
PSMA/FAPI paired imaging / dual-target translational strategy	FAP + PSMA	Exploratory imaging / early translational	Localized high-risk and metastatic PCa	NCT05192694	High translational potential, especially for PSMA-heterogeneous or discordant disease; future therapeutic success will depend on dosimetry and FAPI-PET-based selection.
CXCR4 inhibitor-based approaches	CXCL12/CXCR4	Pilot / early phase	Advanced PCa	Various (e.g., NCT02478125)	Biologically compelling for stromal trafficking and immune exclusion, but future studies should include

	immune-spatial or stromal co-endpoints rather than PSA alone.

Abbreviations: ADT, androgen deprivation therapy; AR, androgen receptor; CXCL12, C-X-C motif chemokine ligand 12; CXCR4, C-X-C motif chemokine receptor 4; FAP, fibroblast activation protein; FAPI, fibroblast activation protein inhibitor; MET, mesenchymal–epithelial transition factor; mHSPC, metastatic hormone-sensitive prostate cancer; NCT, ClinicalTrials.gov identifier; PCa, prostate cancer; PD-L1, programmed death-ligand 1; PSA, prostate-specific antigen; PSMA, prostate-specific membrane antigen; TGF-β, transforming growth factor-beta; VEGFR2, vascular endothelial growth factor receptor 2.

Disclaimer/Publisher's Note: The statements, opinions and data contained in all publications are solely those of the individual author(s) and contributor(s) and not of MDPI and/or the editor(s). MDPI and/or the editor(s) disclaim responsibility for any injury to people or property resulting from any ideas, methods, instructions or products referred to in the content.

https://doi.org/10.3390/cancers18101600
